# Advances in cancer immunotherapy: Strategies and innovations strategies for adoptive immunotherapy of cancer

**DOI:** 10.22038/ijbms.2025.88384.19090

**Published:** 2025

**Authors:** Leila Moeinzadeh, Mohammad-Reza Mahmoudian-Sani, Daryush Purrahman, Fatemeh Azghadi, Mohamad Amin Darbandi

**Affiliations:** 1 Aryogen Pharmed Biopharmaceutical Research Center, Alborz University of Medical Science, Karaj, Iran; 2 Thalassemia and Hemoglobinopathy Research Center, Health Research Institute, Ahvaz Jundishapur University of Medical Sciences, Ahvaz, Iran; 3 Student Research Committee, School of Allied Medical Sciences, Shahroud University of Medical Sciences, Shahroud, Iran; 4 Clinical Research Development Unit, Golestan Hospital, Ahvaz Jundishapur University of Medical Sciences, Ahvaz, Iran

**Keywords:** Adoptive cell transfer, Bispecific antibodies, Chimeric antigen receptor T-cells, Immune checkpoint inhibitors, Immunotherapy, Neoplasm, Tumor microenvironment

## Abstract

Cancer immunotherapy has emerged as a transformative approach in oncology, offering alternatives beyond traditional treatments. This narrative review focuses on major innovations, including adoptive cell therapy (ACT), chimeric antigen receptor T-cells (CAR-T), T-cell receptor (TCR) engineering, monoclonal antibodies (mAbs), bispecific antibodies (BsAbs), and immune checkpoint inhibitors (ICIs). The central aim of this article is to analyze how these technologies improve antitumor responses and help overcome resistance in hematologic and solid tumors. This narrative review combines the latest findings from clinical and preclinical studies to highlight therapeutic potentials and challenges. Key observations include the clinical success of CAR-T cells in treating blood cancers, the expanding application of ICIs in solid tumors, and the evolving structure-function relationship of BsAbs in recruiting immune effectors. This paper concludes by evaluating the current limitations of these immunotherapeutic strategies and discusses future directions for integrating them into personalized cancer therapy.

## Introduction

**Figure 1:**
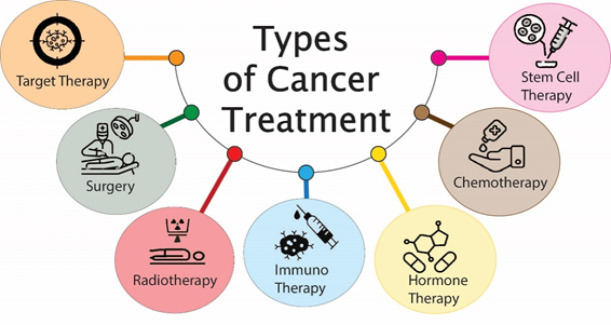
Types of cancer therapy in chronological order from left to right

Cancer is one of the leading causes of death and medical burden worldwide in both developed and developing countries (1). This condition is also one of the most critical obstacles to increased life expectancy in the 21st century (2). The causes of cancer are multifactorial, including aging, population growth, and changes in the prevalence of risk factors (3). Late-stage diagnosis, treatment resistance, and metastasis contribute to poor clinical outcomes in many patients (4, 5). Although traditional therapies, such as surgery, chemotherapy, and radiotherapy** (**Figure 1), have improved survival in some cancers, their effectiveness is often limited by adverse effects, drug resistance, and an inability to control metastatic spread (6). Recent focus has shifted toward understanding the tumor microenvironment (TME), which comprises cancer cells, fibroblasts, endothelial cells (ECs), mesenchymal stem cells (MSCs), and immune components that promote tumor resistance and immune evasion (7). Cancer immunotherapy has emerged as a novel approach that targets the immune system or the TME rather than tumor cells directly (8-10). It utilizes antibodies (Abs), cytokines, dendritic cells, and effector T-cells to activate or restore antitumor immunity (11, 12). However, immune responses are often suppressed due to inhibitory elements within the TME and tumor-induced T-cell dysfunction (13, 14). Checkpoint inhibitors, such as pembrolizumab, nivolumab, and ipilimumab, have revolutionized the treatment of malignancies, including non-small cell lung cancer (NSCLC) and melanoma, by targeting the programmed cell death protein 1 (PD-1) and cytotoxic T-lymphocyte-associated protein 4 (CTLA-4) pathways (15-17). In parallel, vaccines based on peptides, dendritic cells, and oncolytic viruses have also been explored (18-20). Adoptive cell therapy (ACT) involves the *ex vivo* expansion and reinfusion of Tumor-Infiltrating Lymphocytes (TILs), chimeric antigen receptor T-cells (CAR-T), or chimeric antigen receptor-natural killer cells (CAR-NK). CAR-NK cells have demonstrated high response rates in hematologic cancers (21-23). Despite these advances, challenges such as immune evasion, antigen loss, and therapy-associated toxicity persist (24-28). Recent studies have highlighted the promising role of moronecidin-like peptides (MLP) as immunomodulatory agents. In a murine melanoma model, MLP, combined with an anti-PD-1 antibody, significantly enhanced CD8⁺ T-cell responses and improved survival compared to monotherapies, demonstrating synergistic effects in overcoming immune resistance (29). Similarly, in a breast cancer model, a synthetic variant of MLP derived from the Hippocampus not only induced tumor cell apoptosis but also stimulated dendritic cell maturation and CD8⁺ T-cell activation, ultimately reducing tumor burden and prolonging survival (30). These findings support combining immune checkpoint inhibitors (ICIs) with immune-activating peptides to enhance therapeutic efficacy (15-17). Therefore, this review highlights current immunotherapeutic technologies, including CAR-T, CAR-NK, T-cell receptor-modified cells (TCR-modified cells), monoclonal antibodies (mAbs), bispecific antibodies (BsAbs), bispecific T-cell engagers (BiTEs), and ICIs, while discussing their mechanisms, clinical outcomes, challenges, and prospects for combination strategies. The aim is to define their roles in overcoming therapeutic resistance and advancing personalized cancer therapy.

### Immunotherapeutic strategies

The concept of biological response modifiers (BRMs) was first introduced in the 1970s to describe agents capable of modulating the immune system to treat cancer, prevent transplant rejection, and alleviate autoimmune diseases (31). BRMs can either stimulate or suppress immune functions, and some possess direct antitumor properties by inhibiting the growth and invasion of cancer cells. There are two main types of BRMs used in cancer biotherapy: specific and non-specific agents. The particular group includes cancer vaccines, ACT, and various forms of Abs, such as mAbs, single-chain variable fragments (scFvs), BsAbs, and BiTEs. These agents typically elicit antigen-specific immune responses or exert direct cytotoxic effects on tumor cells. The non-specific BRMs (nsBRMs) include checkpoint regulators, cytokines, and immunostimulatory adjuvants such as interleukin-2 (IL-2) and interferons (IFNs), which enhance the overall immune activity without targeting specific antigens. These two BRM categories are often combined to improve therapeutic efficacy **(**Table 1**)** (11, 32). Despite remarkable progress in cancer immunotherapy, significant obstacles such as tumor recurrence and resistance to treatment remain. For instance, many patients eventually relapse due to immune evasion, antigen loss, or the emergence of immunosuppressive tumor microenvironments. Conventional therapies often fail to induce lasting responses in such settings, emphasizing the urgent need for innovative immunotherapeutic interventions. Therefore, identifying novel immune targets and optimizing BRM strategies remain essential for managing patients who are refractory to current therapies or prone to relapse.

### Adoptive cell transfer

ACT has shown significant potential in treating advanced cancers that are typically resistant to conventional therapies, and it is rapidly progressing toward becoming a standard of care (SOC) in oncology (33). In recent years, significant advances in cellular immunotherapy have included the use of stimulating or feeder cells to expand effective immune cells such as NK cells and TILs, along with the development of engineered T-cell receptors (TCRs) and CAR T-cells, which are frequently employed in adoptive cell-based therapies to treat a wide range of malignancies (34, 35). GPRC5D-targeted CAR T-cell therapy has demonstrated promising efficacy in relapsed/refractory multiple myeloma, with an overall response rate of 87% and 65% minimal residual disease (MRD) negativity. Common adverse events included anemia, cytokine release syndrome (CRS), and hypocalcemia, supporting its safety and potential as a valuable component of adoptive cell transfer strategies (36).

### TIL-based strategy

Despite specifically targeting lymphocytes in the TME, TILs often fail to eliminate tumors due to the presence of immunosuppressive agents in the tumor environment (37). To boost their antitumor activity, researchers have cultured and expanded tumor-derived lymphocytes *ex vivo* and reinfused them into patients (25). A significant advantage of TIL therapy over other cell-based immunotherapies is that it does not require genetic modification of the cells. Since patients receive their own expanded TILs, these cells can efficiently recognize and destroy tumor cells. Significant progress is still needed to translate TIL therapy into a practical and standardized cancer treatment. The primary TIL populations include T-cells, B-cells, and NK cells. Although TILs have long been observed, their immunological significance and therapeutic potential have only recently been understood, partly due to technological limitations (37). Recent findings suggest that most TILs target mutant self-proteins rather than well-characterized tumor antigens (38). Nonetheless, *ex vivo* TIL expansion remains problematic, as it is time-consuming and often yields insufficient functional cells for therapy (39, 40).

### CAR T-cell-based strategy

Another significant advance in immunotherapy is the CAR T-cell strategy, which involves genetically modifying autologous T-cells to express synthetic receptors targeting extracellular tumor-associated antigens (TAAs) (41). Unlike traditional T-cell therapies, CAR T-cells do not rely on TCR recognition. Instead, their design enables antigen-specific cytotoxicity, potent *in vivo* activity, and often requires only a single administration (34). CARs are chimeric receptors composed of an extracellular scFv, a transmembrane domain, a costimulatory domain, and immunoreceptor tyrosine-based activation motifs (ITAMs). While structurally distinct from TCRs, CARs bind TAAs on tumor cell surfaces, including proteins, carbohydrates, and gangliosides, and initiate T-cell activation, proliferation, and cytotoxicity (42). Depending on their intracellular domain design, five generations of CARs have been developed (43, 44). Second-generation CARs incorporating CD28 or 4-1BB domains are widely used in clinical trials targeting CD19-expressing B cells in B-cell malignancies (41). Currently, anti-CD19 CAR T-cells are approved for treating ALL, NHL, and CLL. These therapies may use bulk T-cell populations or separated CD4⁺ and CD8⁺ subsets, most often as autologous infusions after apheresis. The engineered cells are reinfused into the same patient to target tumor-expressed antigens (45, 46). To overcome Graft-Versus-Host Disease (GVHD) and Host Versus Graft Rejection (HVGR), universal or allogeneic CAR T-cells (off-the-shelf) have been developed. These cell lines lack endogenous TCRs and MHC-I, making them broadly applicable in cancer research or infectious disease studies. However, ensuring controlled proliferation, avoiding overactivation, and introducing safety switches remain unresolved challenges (47). Many CAR constructs are currently undergoing phase I/II clinical trials, exploring safety and efficacy in various cancers (48). Notably, CAR T-cells have been combined with checkpoint inhibitors to improve therapeutic outcomes (49). However, manufacturing challenges, especially in elderly or chemotherapy-treated patients, and rapid *in vivo* differentiation into short-lived effectors, still limit CAR T-cell efficacy (50). FDA-approved CAR T-cell therapies, such as Kymriah®, Yescarta®, Breyanzi®, Abecma®, and Tecartus®, have demonstrated response rates exceeding 80% in B-cell malignancies, including relapsed/refractory ALL, NHL, and multiple myeloma (51-53). Despite these successes, solid tumors pose significant barriers, including TAA heterogeneity, antigen escape, on-target/off-tumor toxicity, and immunosuppressive TMEs (42). Moreover, CRS and neurotoxicity are frequent complications, ranging from mild flu-like symptoms to life-threatening multi-organ failure (54). Limitations in scalability, accessibility, and virus-associated side effects have also restricted the broader use of CAR T cells (46, 55). To address these challenges, novel approaches have emerged:

• Combining CAR T-cells with other anticancer therapies

• Advanced CAR designs with enhanced persistence and reduced toxicity

Using CRISPR/Cas9 gene editing to knock out immune checkpoints, improve cellular fitness, and generate universal allogeneic CARs (56-58). These CRISPR-modified CAR T-cells, which lack TCR and MHC molecules, reduce the risk of GVHD, enhance accessibility, and lower manufacturing costs. They also demonstrate improved survival and function in hostile tumor environments, offering promise for solid tumors that previously resisted conventional CAR T-cell therapy. Together, these innovations position CAR T-cell and CRISPR-based therapies to revolutionize future cancer treatment paradigms.

### CAR-NK cell-based strategy

**Figure 2:**
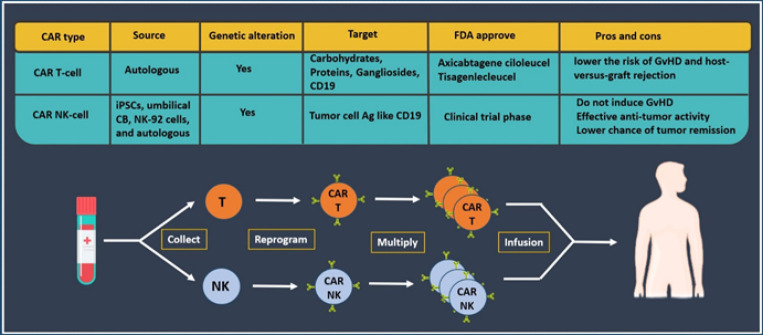
Compression between CAR T-cell and CAR NK-cell

NK cells play crucial roles in limiting cancer progression and metastasis. In the TME, they regulate both adaptive and innate immunity by secreting pro-inflammatory chemokines, which attract additional NK cells to tumor-associated sites (52). These properties make NK cells appealing candidates for chimeric antigen receptor (CAR) engineering, offering several advantages over CAR T-cells. First, allogeneic NK cells do not cause GvHD. Second, their short lifespan allows potent antitumor activity while limiting long-term side effects such as cytopenia. Third, compared to CAR T-cells, CAR-NK cells are less prone to antigen escape because they also kill tumor cells via their natural cytotoxic receptors (59). Despite the remarkable success of CAR T-cell therapies, significant limitations have prompted the development of alternative platforms. CAR-NK cells retain the anticancer efficacy of CAR T-cells while potentially avoiding many of their toxicities, including CRS and neurotoxicity **(**Figure 2**)** (52). In a pivotal study, Liu *et al*. engineered CAR-NK cells from genetically modified cord blood (CB) that express an anti-CD19 CAR, IL-15 for cell persistence, and an inducible caspase-9 (iCasp9) suicide switch to eliminate the cells *in vivo* if needed. Their preclinical studies showed potent *in vivo* lysis of CD19⁺ leukemia cells, prolonged NK cell survival via IL-15 expression, and efficient leukemia clearance following activation of the iCasp9 switch (60). While the development of CAR-NK therapies remains promising, challenges persist with cell isolation, transduction, and expansion. As such, ongoing clinical trials are exploring CAR-NK cells derived from induced pluripotent stem cells (iPSCs) and other progenitor sources (52). For instance, Li et al. generated an iPSC-derived CAR-NK product targeting mesothelin (MSLN), which is highly expressed on several solid tumors (61). Their construct included a 2B4 (CD244) costimulatory domain, CD3ζ activation domain, and an NKG2D transmembrane domain, resulting in enhanced tumor cell lysis. CAR-NK cells are being investigated in multiple Phase I trials for various cancers, including ovarian cancer, glioblastoma, NSCLC, AML, ALL, and other B-cell malignancies. Various NK sources have been employed, such as iPSCs, umbilical cord blood (UCB) NK cells, NK-92 cell lines, and autologous peripheral blood NK cells. Although further safety validation in large cohorts is needed, current data suggest that CAR-NK cells elicit fewer severe toxicities than CAR T-cells. This is likely due to the inherent biological differences between NK and T-cells upon CAR activation (62). CAR-NK therapies integrate innate cytotoxicity with precision targeting, providing MHC-independent immunotherapy. Their success depends on optimal receptor design, target selection, and overcoming TME-associated barriers. Combining CAR-NK cells with complementary immunotherapies or adjuvants may be especially effective in metastatic cancers (63). As research advances, CAR-NK cells represent a powerful next-generation platform, supported by advancements in gene editing and NK cell homing that enhance their therapeutic potential. Preclinical and early clinical results further endorse their promise as alternatives or complements to CAR T-cell therapy (64).

### Monoclonal antibodies

For over two centuries, immunization and antibody-based therapies have played a crucial role in advancing medicine, greatly improving global health. Abs are vital parts of the adaptive immune system, involved in recognizing and neutralizing pathogenic and foreign antigens (65). Although BsAbs are increasingly used in modern immunotherapies, most Ab engineering strategies still preserve the IgG architecture (66). mAbs, which are designed to target a single antigen or tumor-associated growth factor, represent one of the earliest immunotherapeutic tools for cancer. However, their efficacy is often compromised due to immune evasion by tumor cells, leading to resistance. To overcome these limitations, strategies such as combining TAAs with antigen-inexperienced T cells have been proposed (67). mAbs are produced in large quantities for both diagnostic and therapeutic applications (68). In cancer therapy, they can bind to tumor cells and either inhibit their growth, induce apoptosis, or prevent metastasis. They can also be conjugated with drugs, toxins, radioisotopes, cytokines, or other active agents for targeted delivery (11). Additionally, mAbs are often administered alongside chemotherapy to enhance therapeutic outcomes. mAbs are widely used across multiple fields, including anti-thrombotic therapy, antiviral treatment, autoimmune disease management, and oncology. In cancer, specifically, several mAbs have been approved by the US Food and Drug Administration (FDA) (11). Adalimumab, the first mAb derived from phage display, was approved for treating autoimmune diseases (69). Bevacizumab, a humanized anti-VEGF mAb, is used to treat glioblastoma, NSCLC, and metastatic renal cell carcinoma (70). Cetuximab, a chimeric human-mouse mAb targeting the epidermal growth factor receptor (EGFR), is approved for the treatment of colorectal and head and neck cancers (71). Despite their enormous therapeutic potential, mAbs are inherently limited by their single-target specificity, whereas many cancers involve multiple signaling pathways (72). In solid tumors, acquired resistance often results from genetic mutations that alter cell phenotypes, thereby diminishing the efficacy of mAbs (73). Additionally, high interstitial fluid pressure in the TME acts as a physical barrier, reducing the penetration of large macromolecules, such as mAbs (74). As a result, peripheral tumor zones may receive subtherapeutic concentrations, leading to treatment failure and resistance development (75). Therapeutic mAbs are increasingly used to target tumor cells precisely, thereby reducing the systemic toxicity typically associated with chemotherapy (76, 77). Nevertheless, due to their limited efficacy as monotherapies, mAbs are commonly used in combination with chemotherapy (75). Researchers continue to develop novel mAbs targeting surface antigens on brain, lung, breast, ovarian, prostate, colon, and hematologic tumors, including leukemia, lymphoma, and melanoma (11). Currently, more than 500 mAbs are approved or under clinical investigation for autoimmune, hematologic, and malignant disorders, including both solid and hematologic cancers (75, 78). Ultimately, molecularly targeted therapies, particularly mAbs, are at the forefront of precision oncology, offering advantages over traditional treatments by selectively inhibiting critical signaling pathways. These strategies help reduce toxicity and circumvent resistance mechanisms (79).

### Bispecific antibodies

BsAbs are engineered molecules designed to recognize and bind two distinct antigens or epitopes simultaneously. This dual specificity enables BsAbs to either block multiple oncogenic pathways or redirect immune effector cells to tumor sites (80, 81). Their structural diversity and functional versatility have made them highly attractive in the field of oncology. BsAbs can be broadly categorized into Fc-containing (IgG-like) and Fc-free formats. Fc-free constructs such as scFvs, diabodies, triabodies, and tetrabodies formed by linking VH and VL regions with flexible peptide linkers offer superior tumor penetration but suffer from rapid clearance due to short half-lives. In contrast, Fc-containing BsAbs, such as triomAbs, retain Fc-mediated effector functions and benefit from prolonged serum persistence through Fcγ receptor engagement (82-85). The development of BsAbs has progressed through chemical recombination of mAbs and the fusion of hybridomas to create quadromas, which secrete dual-specific antibodies (86). Modern strategies employ recombinant technology (rAbs), enabling efficient production and greater design flexibility (80). Platforms like phage display have further accelerated the generation of large Ab fragment libraries targeting specific tumor-associated antigens (87). BsAbs provide multiple therapeutic advantages. They offer enhanced specificity through simultaneous binding to two TAAs, which minimizes off-target binding (88). Dual pathway inhibition prevents redundancy-driven resistance by blocking multiple signaling routes (81, 88). They also recruit immune effectors, such as T cells and NK cells, to tumor sites to amplify cytotoxic responses (89, 90). By modulating two functional axes, BsAbs help delay or prevent tumor escape mechanisms (90). Their design flexibility supports crossing the blood-brain barrier, extending serum half-life, and enabling pre-targeting strategies (81, 88). From a manufacturing perspective, BsAbs improve production efficiency by reducing time, cost, and ethical concerns compared to dual mAb therapies (91, 92). BsAbs redirect immune effectors via MHC-independent mechanisms, typically by binding CD3 on T cells and a second TAA such as CD19, CD20, CD33, CD123, EpCAM, or HER2, thereby forming a cytolytic immune synapse (89, 93, 94). This approach has proven particularly effective in hematologic malignancies, such as leukemia and lymphoma, where BsAbs demonstrate high efficacy due to the accessibility of circulating tumor cells (95). Despite their promise, BsAbs face several limitations and challenges. Fc-free molecules, such as BiTEs, exhibit short half-lives, requiring continuous infusion (96). Steric hindrance may restrict access to epitopes in solid tumors, and immunogenicity, aggregation, low expression yields, and reduced stability can hinder clinical application (89). Specificity remains critical, as many TAAs (e.g., CD33, HER2, CEA) are also expressed, albeit at lower levels, in normal tissues, which increases the risk of on-target/off-tumor toxicity (97, 98). BiTEs are a subclass of BsAbs composed of two scFvs, one targeting CD3 on T cells and the other a TAA on tumor cells. This structure forms a cytolytic synapse, activating T cells and triggering the release of perforin and granzyme B to induce apoptosis. Notably, this occurs independently of MHC, TCR specificity, or costimulatory signals, making BiTEs effective even in immune-evasive tumor environments (80, 99, 100). Multiple BsAbs have received FDA approval, including Blinatumomab (CD19/CD3), Amivantamab (EGFR/MET), Teclistamab-cqyv (BCMA/CD3), Epcoritamab (CD20/CD3), and Tebentafusp (gp100/CD3) (101). Although Catumaxomab (EpCAM/CD3) was approved earlier, it was later withdrawn due to commercial reasons; however, it played a crucial role in validating BsAb therapeutic concepts (102). Among hematologic malignancies, such as ALL and DLBCL, blinatumomab has demonstrated robust efficacy through polyclonal T cell redirection (103, 104). However, BsAbs, especially BiTEs, can induce immune-related toxicities, such as CRS, neurotoxicity, and hypersensitivity reactions (105-108). CRS, characterized by elevated cytokines (e.g., IL-6 and TNF-α), may require corticosteroids or tocilizumab. Other side effects include cytopenias, liver toxicity, and infection risks, underscoring the need for optimal dosing and careful TAA selection. Looking forward, next-generation BsAbs are being developed with extended half-lives, improved tumor selectivity, and immune modulation capabilities. Delivery systems such as BsAb-expressing MSCs are under investigation to target therapy and reduce systemic exposure (109, 110). Additionally, combining BsAbs with ICIs or tumor-penetrating peptides, such as moronecidin-like agents, may overcome resistance and expand therapeutic potential, particularly in solid tumors.

### Immune checkpoint therapy

ICIs are a class of mAbs that potentiate T-cell-mediated antitumor responses by blocking inhibitory receptors or their ligands, notably cytotoxic CTLA-4, programmed death-1 (PD-1), and programmed death ligand-1 (PD-L1) (111). Their introduction has significantly reshaped the therapeutic landscape of several malignancies, including melanoma, NSCLC, and renal cell carcinoma (112, 113). These checkpoints serve as immunological “brakes” that tumors exploit to evade immune destruction. By inhibiting these pathways, ICIs restore T-cell activity against malignant cells. However, a substantial proportion of patients fail to respond due to primary or acquired resistance, stemming from mechanisms such as poor tumor immunogenicity, absence of TILs, or compensatory activation of alternative immune checkpoints (114). Moreover, ICIs can lead to immune-related adverse events (irAEs), including colitis, hepatitis, endocrinopathies, and pneumonitis, which can limit their clinical applicability (115). Several factors, including tumor mutational burden, gut microbiota, and host genetics, influence response heterogeneity. To date, over 100 ICIs have entered clinical development or received regulatory approval (116). Notable agents include anti-PD-1 antibodies (e.g., nivolumab and pembrolizumab), anti-PD-L1 antibodies (e.g., atezolizumab, avelumab, durvalumab), and combination regimens such as nivolumab plus ipilimumab, which have demonstrated superior efficacy in some cancers but at the cost of increased toxicity (117). Cadonilimab (AK104) represents an emerging bispecific antibody that simultaneously targets PD-1 and CTLA-4, offering enhanced dual checkpoint blockade within a single molecule (118). This engineered approach may balance immune activation and toxicity by modulating binding affinity and Fc-effector functions. Preclinical and clinical findings indicate that Cadonilimab can overcome resistance observed with monotherapies, providing sustained immune activation with an acceptable safety profile. Checkpoint blockade has undoubtedly revolutionized cancer immunotherapy. However, limitations remain in terms of variable patient responses, toxicity management, and the development of predictive biomarkers (119).

### CTLA-4 therapy hindering T-cell costimulatory signal

CTLA-4 is a critical immune checkpoint expressed on activated T cells, T regulatory cells (Tregs), and B cells, acting as a negative regulator of T-cell activation by binding to CD80 and CD86 on antigen-presenting cells. This interaction competes with the costimulatory receptor CD28, thereby attenuating T-cell responses (120, 121). Blocking CTLA-4 restores effective costimulatory signaling and promotes antitumor immunity. Emerging evidence also implicates antibody-dependent cellular cytotoxicity (ADCC) in selectively depleting intra-tumoral Tregs, contributing to the therapeutic effects of anti-CTLA-4 antibodies (122). Ipilimumab, the first FDA-approved CTLA-4 inhibitor, demonstrated a significant survival advantage in metastatic melanoma, marking a pivotal advancement in immunotherapy (75). By antagonizing CTLA-4, ipilimumab enhances T-cell activation, suppresses Tregs, and augments the recognition of TAAs. Clinical trials, including a phase II study in NSCLC, have confirmed its efficacy (40). Beyond melanoma, CTLA-4 blockade is under investigation across multiple tumor types. For instance, in metaplastic breast cancer, dual therapy with anti-PD-1 and anti-CTLA-4 antibodies produced an overall response rate (ORR) of 12%, with a 12-month median overall survival in ongoing phase II trials (123). Mechanistically, CTLA-4 inhibition activates CD8+ effector T cells and diminishes Treg-mediated immunosuppression, thereby fostering robust antitumor responses. Additional molecules, such as soluble CTLA-4, may further influence therapeutic outcomes and warrant consideration as potential biomarkers (124). In summary, CTLA-4-targeting ICIs exert multifaceted effects on the immune landscape, offering substantial benefit in selected patients. Their optimal use requires an understanding of immune contexture, resistance mechanisms, and combination strategies to achieve durable responses.

### PD-1/PD-L1 therapy hindering TCR signaling

The PD-1 receptor and its ligands, PD-L1 and PD-L2, constitute a crucial immune checkpoint pathway that regulates T-cell activation, peripheral tolerance, and exhaustion (125). Tumor cells often exploit this axis by overexpressing PD-L1, thereby suppressing the activity of cytotoxic T lymphocytes (CTLs) and evading immune surveillance (126). Upon PD-1 engagement, SHP-2 phosphatase is recruited to its cytoplasmic ITSM domain, leading to the dephosphorylation of TCR signaling molecules such as CD3ζ and ZAP70, which attenuates TCR signaling and cytokine production (127). Consequently, effector T cells within the TME become functionally inactivated or “exhausted”. Multiple downstream signaling cascades are disrupted through PD-1 activation, including the PI3K/Akt, MAPK/ERK, and JAK/STAT pathways (128). These changes impair glucose metabolism, cell proliferation, and cytokine gene transcription, ultimately diminishing antitumor immunity. Resistance to anti-PD-1/PD-L1 therapy may arise from intrinsic tumor factors, such as PTEN loss, β-catenin signaling, and VEGF-mediated immune exclusion, as well as from adaptive feedback, in which inflammatory cytokines, like IFN-γ, up-regulate PD-L1, thereby reinforcing immunosuppression (129). Mechanistically, after antigen presentation via MHC-TCR interaction, tumor-infiltrating T cells release IFN-γ, which further induces PD-L1 expression on tumor and stromal cells (130, 131). This creates a negative feedback loop, limiting T-cell function in the TME (132). PD-1 contains ITIM and ITSM motifs that, once phosphorylated, recruit SHP-2 to inhibit key signaling molecules. This suppresses IL-2 secretion, glucose uptake, and cell survival pathways (133), rendering effector T cells less capable of mediating cytotoxicity (134, 135). Significantly, PD-1/PD-L1 engagement also contributes to immune tolerance by promoting the differentiation of naïve CD4⁺ T cells into FOXP3⁺ regulatory T cells, independent of TGF-β, as shown in both *in vivo* and *in vivo* models (111, 136). This dual mechanism directs T-cell inhibition and Treg induction, thereby reinforcing immune escape and tumor progression. Clinical studies have validated the therapeutic benefit of PD-1/PD-L1 blockade in malignancies such as metastatic melanoma, NSCLC, renal cell carcinoma, bladder, and head and neck cancers (137, 138). Anti-PD-1 agents (nivolumab, pembrolizumab) and anti-PD-L1 agents (atezolizumab, durvalumab, avelumab) have all received regulatory approval for various indications (139). These agents have shown improved overall survival and durable responses, often outperforming conventional therapies with a more favorable toxicity profile compared to CTLA-4 inhibitors (120, 140, 141). Despite these successes, not all patients respond to treatment. Hence, combination therapies are under investigation. For example, PD-1/PD-L1 blockade has been shown to re-sensitize tumors to BiTE therapies, such as AMG330 (anti-CD33×CD3), by restoring T-cell cytotoxicity (126, 127). Additionally, dual treatment with checkpoint inhibitors and BsAbs has demonstrated enhanced efficacy in colorectal cancer and B-cell lymphoma, as evidenced by increased immune activation and tumor regression in preclinical and early clinical trials (128, 129, 142). Combining PD-1/PD-L1 inhibitors with radiation, chemotherapy, or T-cell engagers (e.g., anti-CEA×CD3) offers a promising strategy to remodel the TME, reduce MDSCs, and enhance infiltration of TILs (115, 116). Furthermore, novel anti-PD-L1 agents, such as atezolizumab and durvalumab, have been engineered with Fc-silent mutations to minimize complement-dependent cytotoxicity (CDC) and ADCC, thereby improving their safety profiles (143). Ongoing clinical trials are exploring innovative combinations, personalized biomarker strategies (e.g., PD-L1 expression, TIL density), and next-generation ICIs to overcome resistance and broaden patient benefit (125, 144). Ultimately, targeting the PD-1/PD-L1 axis remains a cornerstone of immuno-oncology, with continued refinements poised to enhance therapeutic efficacy across malignancies. 

### Bispecific T-cell engager

BiTEs are recombinant, engineered proteins designed to physically link CTLs to tumor cells, thereby promoting direct immune-mediated tumor destruction. These molecules typically consist of scFvs: one that recognizes CD3 on T cells and the other that targets a TAA on cancer cells. In an innovative approach, a novel CD3/PD-L1 BiTE was developed by genetically fusing the VL and VH chains of an anti-PD-L1 antibody to those of an anti-CD3 antibody. This format facilitates the redirection of T cells, including CD8⁺, CD4⁺, and CD3⁺ NKT cells, as well as L1 PD-L1-expressing tumor cells, thereby overcoming PD-1 axis–mediated immunosuppression. *In vivo* experiments demonstrated the robust and selective activation of healthy donor-derived T cells, suggesting that this CD3/PD-L1 BiTE may serve as a potent immune activator in patients with PD-L1-positive solid tumors. Notably, its most excellent efficacy was observed when combined with immunotherapeutic agents that do not directly counteract PD-1–mediated immune inhibition (145). This bispecific construct holds promise not only due to its ability to bypass immune evasion mechanisms but also because it bridges the immunological synapse between T cells and tumor cells, facilitating efficient tumor cell killing. Since not all tumor cells uniformly express PD-L1, the synergy of BiTEs with checkpoint inhibitors or other immunotherapies can broaden their therapeutic utility. Among FDA-approved BiTEs, blinatumomab is a well-established prototype that simultaneously targets CD19 on B cells and CD3 on T cells. It effectively mediates B-cell lysis in ALL through T-cell redirection (146, 147). Similarly, teclistamab targets B-cell maturation antigen (BCMA) on myeloma cells and CD3 on T cells, demonstrating potent efficacy in relapsed/refractory multiple myeloma (RRMM) (148-150). Another example, Tebentafusp, utilizes a TCR-like molecule that recognizes gp100, a melanoma-associated antigen, and is linked to an anti-CD3 scFv. This construct enhances antigen-specific recognition and lysis of gp100-expressing melanoma cells (151). Despite their therapeutic potential, BiTEs and other BsAbs present notable risks, including CRS, neurotoxicity, and on-target/off-tumor effects. These toxicities necessitate vigilant clinical monitoring, dose optimization, and supportive care to mitigate adverse events (152). Nevertheless, with proper management, BiTEs remain a transformative class of agents in T cell–redirecting immunotherapies, capable of overcoming immune resistance and broadening cancer treatment options.

### Toxicity associated with BiTE

CRS is one of the most common and severe side effects related to BiTE therapy. It results from the rapid release of cytokines by activated T cells. Symptoms can range from mild, flu-like signs to severe reactions, including high fever, hypotension, and organ dysfunction. Neurological side effects may also occur, such as confusion, seizures, or encephalopathy. Depending on severity, these conditions require close monitoring and proper management. Infections and bleeding can occur due to cytopenia associated with BiTE therapy, including thrombocytopenia and neutropenia. Patients may also experience hypersensitivity reactions, ranging from mild rashes to severe anaphylaxis. The specific BiTE agent and the cancer type being treated can lead to organ-specific toxicities, such as liver toxicity or pulmonary complications (105-107). Close monitoring of vital signs and laboratory parameters is crucial, especially during the early phases of treatment. Supportive care includes hydration and antipyretics. In severe cases of CRS or neurological toxicity, corticosteroids may help diminish the inflammatory response. An IL-6 receptor antagonist, tocilizumab, can counteract cytokine release in severe CRS.

**Table 1:** Comparison of characteristics between ICI, CAR T-CELL, and BiTE therapy (157-159)

	ICI	CAR T-CELL	BiTE
Structure	Monoclonal antibody targeting the immune checkpoint protein	A synthetic T cell construct encoding a scFv against a tumor antigen linked to activation and costimulatory motifs	A recombinant protein composed of two linked scFvs; one binds to CD3 on T cells and the other to target a tumor antigen on tumor cells
Antitumor mechanisms	Blocking the inhibitory immune checkpoint proteins that result in cytotoxic T cell-mediated immune response and restoring immune system function	Inducing tumor cell lysis by the formation of immune synapses between T cells and tumor cells	Inducing tumor cell lysis by the formation of immune synapses between T cells and tumor cells
Recruitment of T cells	Passive, acting on tumor-infiltrating and endogenous T cells to kill tumor cells	Active, redirecting engineered T cells outside of the body to kill tumor cells	Passive, dependent on endogenous T cells, and redirecting them to kill tumor cells
Production and availability	Hybridoma technology is readily available for all patients, providing immediate (“off-the-shelf”) benefits	Genetically engineering a patient’s T cells outside the body, individualized for each patient, is a time-consuming process (weeks for autologous CAR-T cells)	Genetically engineered and purified from mammalian cell lines, it is effective for all patients and is immediately available, making it readily accessible (“off-the-shelf”)
Indications	Mainly in solid tumors, with approval in a small part of hematologic neoplasms	All in hematologic neoplasms	All in hematologic neoplasms and some solid tumors
Toxicity	Hyperactivation and Hypersensitivity	CRS, neurotoxicity	CRS, neurotoxicity
Advantages	Broad-spectrum antitumor activity, easy production	MHC-independent, TCR-independent, endogenous T cell-independent	MHC-independent, TCR-independent, relatively easy production, tumor-infiltrating T cell-independent
Disadvantages	Tumor-infiltrating T cell-dependent, immune checkpoint expression-dependent, MHC-dependent, TCR-dependent, drug resistance	Lack of efficacy for solid tumors, long-term and complex production, antigen-dependent, on-target off-tumor effects, and targeting multiple antigens.	Antigen-dependent, continuous administration due to a short half-life, on-target off-tumor effects
MHC Dependent	YES	NO	NO
CD3 engagement	Variable	scFv-CD3ζ	scFv-CD3ε
Tumor penetration	Better with small molecules	Worse	Better with small molecules
Half-life	Variable	It might be extended with memory immunity (even years)	Variable (short)
Effector cell	Variable	*ex* * vivo* engineered CD8+ and CD4+ T cells	Unmanipulated T cells (Endogenous CD8+ and CD4+ T cells)
Dosing	Repeat dosing	Single infusion (“one-shot”)	Repeat dosing
FDA approval	Ipilimumab (CTLA-4), Nivolumab (PD-1), Atezolizumab (PD-L1), Avelumab (PD-L1)	Tisagenlecleucel (CD19), Idecabtagene Vicleucel (BCMA), Ciltacabtagene Autoleucel (BCMA)	Catumaxomab (EpCAM), Blinatumomab (CD19), Tebentafusp (gp100 peptide-HLA)

ICI: immune checkpoint inhibitor, CAR: chimeric antigen receptor, BiTE: bispecific T cell engager, CRS: cytokine release syndrome, MHC: major histocompatibility complex, TCR: T-cell receptor, scFv: single-chain fragment variable; EpCAM: Epithelial cell adhesion molecule

### Cancer resistance to adoptive immunotherapy

Immune-related resistance remains one of the major obstacles in cancer treatment. This resistance arises from various factors, including host-related, tumor-intrinsic, and TME variables. Tumor-intrinsic mechanisms involve disruptions in antigen presentation pathways, such as the proteasome, transporters, and MHC, as well as alterations in antitumor immune response pathways, including aberrant production of tumor antigens. Additionally, tumor cells within an immunosuppressive TME release inhibitory molecules like PD-L1 and exhibit functional genetic mutations in key pathways such as PTEN/PI3K, CDK4–CDK6, MAPK, EGFR, and KRAS. Metabolic modifications also contribute to resistance, including hypoxia, IDO activity, and the production of adenosine. Alterations in signaling pathways, such as the interferon-γ pathway, further promote immune evasion. In the TME, suppressive immune cells and molecules, including MDSCs, Tregs, TAMs, PD-L1, and CTLA-4, as well as abnormal neovascularization, collectively contribute to resistance. Host-related factors, such as gender, age, body fat composition, and gut microbiota, also influence treatment resistance. Resistance is categorized into three types: Primary resistance (no response from the start), Adaptive resistance (emerges during therapy), and Acquired resistance (relapse after an initial response). Overcoming these challenges requires the identification of predictive biomarkers, the development of personalized treatment strategies, and combination therapies that target multiple resistance pathways (153-156). 

## Conclusion

Recent advances in immunotherapeutic modalities, including BsAbs, ICIs, ACT, and cancer vaccines, have significantly transformed the cancer treatment paradigm. Each strategy contributes distinct advantages in enhancing antitumor immunity. However, challenges such as immune evasion, treatment resistance, toxicity, and patient heterogeneity persist, hindering long-term efficacy. Dual-targeting constructs, such as bispecific formats and agents like Cadonilimab, offer promise by enhancing immune activation while reducing overlapping toxicities. However, no single strategy has demonstrated universal effectiveness. As a result, future directions will depend on rational combination therapies. These may include ICIs integrated with tumor-targeted peptides (e.g., moronecidin-like agents), CAR-T cells combined with checkpoint inhibitors, or BsAbs used in conjunction with personalized tumor vaccines. Furthermore, identifying predictive biomarkers, improving drug delivery systems, and modulating TME will be essential for optimizing outcomes. Ultimately, a deeper mechanistic understanding of immune tumor interactions, alongside the design of tailored immunotherapeutic platforms, holds the key to achieving durable clinical responses and expanding the benefits of immunotherapy across diverse patient populations.

## Declaration

This manuscript was developed with the help of generative artificial intelligence tools. Specifically, the latest version of OpenAI’s ChatGPT was used to improve language, refine the text, and generate summaries. These tools were only used to enhance the manuscript’s clarity, coherence, and readability.
